# Use of baloxavir as adjunctive antiviral therapy to neuraminidase inhibitors in severely immunocompromised individuals infected with influenza

**DOI:** 10.1128/aac.01659-25

**Published:** 2026-03-27

**Authors:** Pauline Vetter, Ana Rita Goncalves Cabecinhas, Manuel Schibler, Laurent Kaiser, Karen Cruz-Fauquex, Yves Chalandon, Stavroula Masouridi-Levrat, Dionysios Neofytos

**Affiliations:** 1 Geneva Center for Emerging Viral Diseases, Geneva University Hospitals (HUG) 27230 , Geneva, Switzerland; 2 Division of Infectious Diseases, Department of Medical Specialties, Geneva University Hospitals 27230 , Geneva, Switzerland; 3 Laboratory of Virology, Diagnostic Department, Geneva University Hospitals 27230 , Geneva, Switzerland; 4 National Reference Center of Influenza, Geneva University Hospitals 27230 , Geneva, Switzerland; 5 Pharmacy, Geneva University Hospitals 27230 , Geneva, Switzerland; 6 Division of Hematology, Department of Oncology, Geneva University Hospitals 27230 , Geneva, Switzerland; Chinese Academy of Medical Sciences & Peking Union Medical College, Beijing, China

**Keywords:** influenza, oseltamivir, baloxavir, treatment, immunosuppression, combination therapy, HCT, mortality, complications, safety

## Abstract

Immunocompromised patients are at risk of developing severe influenza, with protracted viral shedding and development of resistance-associated mutations under antiviral treatment. We report a case series of severely immunocompromised hematology patients, including allogeneic hematopoietic cell transplantation (HCT) recipients, treated with both baloxavir and oseltamivir and describe clinical and virological outcomes and the safety profile of prolonged combination therapy. Allogeneic HCT recipients with influenza infection treated with baloxavir were retrieved via institutional databases. All hospitalized allogeneic HCT patients treated with a combination therapy of baloxavir and oseltamivir over five influenza seasons between October 2019 and May 2025 were included. Six influenza-infected hematology patients (5/6 allogeneic HCT recipients) were treated with combination therapy of oseltamivir and baloxavir. All patients presented with lower respiratory tract infections. Oseltamivir treatment duration ranged from 5 to 31 days, and the number of administered baloxavir doses ranged between one and five. Baloxavir administration was well tolerated, and no adverse events could be attributed to the administered antiviral treatment. All-cause mortality at 3 months post-infection was 66% (4/6), mainly driven by underlying disease. In two patients with protracted shedding, combination therapy did not prevent the development of resistance mutation(s). Combination treatment with prolonged courses of oseltamivir and repeated doses of baloxavir was well tolerated. No definitive conclusions on the efficacy of this approach could be drawn from this study. More data are required on the best treatment of hematology patients infected with influenza.

## INTRODUCTION

Immunocompromised patients are particularly at risk of developing severe disease after influenza infection (1–5). Up to one-third of infected patients following hematopoietic cell transplantation (HCT) may suffer from pneumonia (6). Complicated disease requiring transfer to the intensive care unit (ICU) is observed in up to 10% of cases, with a mortality risk reaching up to 13% at 6 months (6). In addition, immunosuppression is associated with prolonged viral shedding, with a higher risk of developing resistance-associated mutations when antivirals are administered (7). Few antivirals are widely available against influenza viruses. Neuraminidase inhibitors (NAIs), among which oseltamivir is the most frequently used due to its oral administration, block the release of new virions and prevent infection by impeding neuraminidase (NA) cell attachment (8). Baloxavir marboxil (baloxavir) inhibits replication by blocking the endonuclease activity of the viral polymerase acidic (PA) protein (9). Adamantans are no longer in use since the 2000s due to a dramatic rise in resistance (10). While the WHO guidelines on clinical management of influenza recommend baloxavir as the first-line treatment in high-risk patients presenting with non-severe disease, a warning of caution about its use has been emitted in immunocompromised patients due to the risk of acquisition of resistance mutations (11, 12). In patients requiring hospitalization, oseltamivir is still the first-line therapy, given the scarcity of existing data regarding baloxavir use in this context. While combination therapy could theoretically increase clinical effectiveness and limit the emergence of antiviral resistance, it did not demonstrate a clinical benefit in a trial, in which <10% of the participants were immunocompromised, comparing NAI monotherapy to NAI plus baloxavir in patients with severe disease (13). *In vivo*, only a few data exist to support combination therapy of two different antiviral classes in immunocompromised individuals (14).

Since 2019, we have changed our institutional protocol for the treatment of influenza in highly immunocompromised hematology patients, including allogeneic HCT recipients, to combination treatment with oseltamivir and baloxavir. We report a case series of allogeneic HCT recipients treated with a combination therapy of baloxavir and oseltamivir in our institution and detail clinical and virological outcomes, as well as the safety profile of the prolonged combination therapy.

## MATERIALS AND METHODS

### Study design and setting

All allogeneic HCT patients infected with influenza, who were hospitalized in our institution between October 2019 and May 2025 and treated with a combination therapy of oseltamivir and baloxavir, were included in this study.

### Data collection

Allogeneic HCT recipients were identified through the institutional Bone Marrow Transplant Unit database. Baloxavir prescription was retrieved through the hospital pharmacy database. Demographic data, underlying medical conditions, type and duration of treatment administration, influenza type and length of viral shedding, viral and bacterial co-infections, and clinical outcomes have been retrospectively retrieved from medical charts. Influenza type, subtype, and presence of resistance mutations, when available, were collected from the virology laboratory database. Day 0 was considered as the day of influenza symptoms’ onset, or the day of diagnosis when the former was not available.

### Virology procedures

Viral RNA was extracted from nasopharyngeal swabs (NPSs) or bronchoalveolar lavage preserved in viral transport media using the QiaSymphony magnetic-particle system (Qiagen, Basel, Switzerland) according to the manufacturer’s instructions. The extracted genetic material was used for influenza A and B screening using a customized real-time (reverse transcription)-PCR (RT-PCR) respiratory viruses’ panel produced by Eurogentec (Kaneka Corporation, Belgium). One-step reverse transcription and amplification reactions were performed on a Quantstudio 5 thermocycler. Samples were considered positive if an amplification curve was present with a cycle threshold (Ct) value of ≤37. Resistance mutations to oseltamivir and baloxavir were assessed by Sanger sequencing of the NA gene and partial PA gene (around 900 bp, a region covering all substitutions known to be associated with reduced susceptibility). Sequence editing, screening for, and interpretation of the amino acid substitutions associated with decreased susceptibility to both antivirals were performed using SmartGene Integrated Database Network System Influenza Module algorithms (https://apps.idns-smartgene.com/apps/Login.po) based on the WHO summary tables of amino acid substitutions associated with decreased susceptibility to oseltamivir or baloxavir marboxil (15).

### Definitions

Influenza infection was defined as testing positive for influenza by RT-PCR on upper or lower respiratory sampling. Lower tract respiratory infection (LRTI) was defined by the presence of a pulmonary infiltrate on computed tomography (CT) scan (16).

### Antiviral dosage

Oseltamivir was administered orally, and standard doses were adapted, depending on renal clearance, according to the manufacturer’s instructions. Each administered baloxavir dose was adapted to the patient’s weight: <80 kg: 1 tablet of 40 mg PO, ≥80 kg: 80 mg of baloxavir marboxil (Xofluza) and given orally.

## RESULTS

Over the study period, six patients were treated in our institution with baloxavir, all in combination with oseltamivir. All patients were hospitalized, severely immunocompromised due to their underlying hematological disease, and had an LRTI. Patient characteristics are presented in Table 1. Virological characteristics and resistance mutation emergence under antiviral treatment, when available, are presented in Table 2. Most (5/6) patients were infected during the 2023/2024 and 2024/2025 influenza seasons, mostly with influenza A (4/5 available results). Oseltamivir treatment duration ranged between 5 and 31 days, and the number of doses of baloxavir administered ranged between one and five, administered every 72 to 96 hours (Table 2).

**TABLE 1:** Main clinical characteristics of the patients and evolution of influenza disease^*a*^

Patient	Age	Sex	BMI	Vaccination status	Underlying disease	Immunodeficiency scoring index	Infection site	Outcome	Co-infection
1	56	F	19	Unknown	Biphenotypic B/T ALL with CNS infiltration, diagnosed 3 months prior to influenza infection	11	LRTI	Death on D31 from underlying disease	Probable pulmonary aspergillosis (D−61) Urinary tract *Escherichia coli* ESBL infection (D−2)
2	63	M	24	Vaccinated	Persistent bicytopenia post-haploidentical allogeneic HCT for primary idiopathic myelofibrosis 14 months prior to influenza infection, chronic liver GVHD (D −360)	4	LRTI	Discharged with resolution of influenza infection	*Klebsiella pneumoniae* bacteremia (D5) and *Staphylococcus aureus* pneumonia (D6)
3	72	M	22	Unknown	Allogeneic HCT from HLA-incompatible related donor for CML 13 months prior to influenza diagnosis, thrombotic microangiopathy with pancytopenia	3	LRTI	Death on D78 from underlying disease	*Providencia rettgeri* bacteremia (D−13 and D20)
4	59	M	30	Unknown	Allogeneic HCT from MUD for polycythemia vera 6 months prior to influenza infection, with AML relapse	9	LRTI	Death on D107 from underlying disease, after influenza clearance	SARS-CoV-2 infection (D−26) Respiratory syncytial virus infection (D−7): treated with ribavirin from D−5 to D19, immunoglobulin, palivizumab D21 *Clostridioides difficile* colitis (D−4) Possible pulmonary fungal infection (D26)
5	45	F	25	Vaccinated	Second allogeneic HCT from MUD 26 months previously for ALL, relapse 1 month before influenza infection; GIT GVHD	8	LRTI	Death on D14 from severe influenza	Probable pulmonary aspergillosis (D5) Suspected pulmonary bacterial superinfection (D5)
6	72	M	24	Vaccinated (prior season)	Allogeneic HCT matched unrelated donor for AML for the previous 30 months, chronic cutaneous GVHD (D-302)	3	LRTI	Resolution of influenza infection	Colonization with *Mycobacterium avium* (on diagnosis day, D0)

^*a*^ ALL, acute lymphocytic leukemia; AML, acute myelogenous leukemia; BMI, body mass index; CML, chronic myeloid leukemia; CNS, central nervous system; D, day; ESBL, extended-spectrum beta-lactamase; F, female; GIT, gastrointestinal tract; GVHD, graft versus host disease; HCT, hematopoietic cell transplant; ICU, intensive care unit; M, male; MUD, matched unrelated donor; LRTI, lower respiratory tract infection.

**TABLE 2:** Virological characteristics of influenza infections^*a*^

Patient	Influenza season	Influenza type/subtype	Treatment initiation D^*b*^	Oseltamivir	Baloxavir doses	Length of viral shedding^*c*^	Ct values^*d*^	Resistance testing	Effect of the mutation
1	2024/2025	IA/H1N1	1	D1 to D20 (21D)	5 on D1, D4, D7, D10, and D13	8D	D-2: ND D0: Ct = 29 D4: Ct = 30 D8: ND		
2	2024/2025	IA/H1N1	5	D5 to D14 (10D)	2 on D6 and D11	NA, >4D	D5: POS D6: Ct = 24 D9: Ct = 27		
3	2023/2024	IA/H1N1	1	D1 to D10 D33 to D43 D52 to D61 (31D)	4 on D52, D55, D58, and D62	NA, >75D	D-7: NEG POS D33: Ct = 28 D44: Ct = 20 D47: Ct = 17 D51: Ct = 19 D58: Ct = 26 D65: Ct = 31 D75: Ct = 22	D33: 223R D44: 247G	223R: reduced inhibition to oseltamivir, zanamivir, and peramivir 247G: Reduced inhibition to oseltamivir
4	2023/2024	IA/H3N2	1	D1 to D10D19 to D38 (30D)	3 on D3, D17, and D23	35D	D-7: NEG D0: Ct = 20 D2: Ct = 22 D6: Ct = 29 D10: Ct = 29 D13: Ct = 29 D16: Ct = 14 D20: Ct = 29 D23: Ct = 29 D29: Ct = 20 D35: NEG	D0: no resistance D13: no resistance D16: I38T D29: I38T and R292K	I38T: 20-614 EC50 fold change to baloxavir R292K: highly reduced inhibition to oseltamivir, zanamivir, and peramivir
5	2022/2023	IB	5	D5 to D11 (7D)	1 on D6	NA	D5: POS D6: Ct = 22 D7: Ct = 24 (BAL)		
6	2019/2020	IA/NA	NA	D1 to D4 after diagnosis (5D)	1 on D2 after diagnosis	NA	On diagnosis: Ct = 38 (BAL) NEG on NPS the D prior		

^*a*^ BAL, bronchoalveolar lavage; Ct, cycle threshold; D, day; NA, not available; ND, not detected; NEG, negative; NPS, nasopharyngeal swab; POS, positive.

^*b*^ Day of treatment initiation after symptom onset.

^*c*^ From first positive to first negative.

^*d*^ On upper respiratory tract sampling: NPS, unless specified otherwise.

### Case summary

A summary of the clinical history of six patients is available below.

Patient 1 was diagnosed with acute biphenotypic lymphoid leukemia, received the first induction cycle of chemotherapy 3 months before influenza infection, and was treated for a probable invasive pulmonary aspergillosis (IPA) with isavuconazole on day −60. The patient also had unilateral renal lithiasis complicated by pyelocaliceal dilatation requiring ureteral catheterization. Upon admission for HCT and while asymptomatic, routine screening for respiratory viruses was negative. Two days later, after the beginning of conditioning with fludarabine and total body irradiation, the patient developed acute onset of fever, upper respiratory symptoms, and new oxygen requirement. Nasopharyngeal swab was positive for influenza A. A CT scan revealed excavation of the pre-existing known lung infiltrate attributed to IPA, without any new lesions. Under treatment with oseltamivir and baloxavir (five doses every 72 hours), fever subsided, and the inflammatory markers decreased. Combination therapy was well tolerated. The patient required a short stay in the ICU for normocapnic hypoxemic respiratory distress attributed to the sepsis and fluid overload, without need for intubation. Despite clearance of the influenza infection, considering the severity of the underlying hematological disease, a decision was made with the patient not to pursue HCT, and the patient was transferred to the palliative care unit and died 31 days post-influenza diagnosis.

Patient 2 had an allogeneic HCT for primary idiopathic myelofibrosis and developed a possible IPA treated by isavuconazole and liver graft versus host disease (GVHD). While still on high-dose steroids, the patient was hospitalized 14 months post-transplant with hypoxemic respiratory distress 5 days after the beginning of cough and fever. Influenza A (H1N1) infection was confirmed on NPS sampling and was complicated by a bacteremic pulmonary superinfection with K*lebsiella pneumoniae*. The clinical and biological progression was quickly favorable with meropenem and antiviral combination therapy of oseltamivir and baloxavir. Since influenza A virus was still detected on NPS after day 4 of treatment, a second dose of baloxavir was administered. The patient was eventually discharged after an 8-day hospital stay and a total duration of oseltamivir treatment of 10 days. No subsequent testing was available to confirm influenza clearance, but the symptomatology had completely resolved.

Patient 3 received an allogeneic HCT for chronic myeloid leukemia and, despite a complete hematological remission, required a 10-month hospitalization due to several complications before being transferred to our institution. Thirteen months post-transplant and 31 days prior to transfer to our institution, the patient presented with an upper respiratory syndrome and tested positive for influenza A on NPS. He received a 10-day treatment course of oseltamivir. The patient’s course was further complicated by febrile neutropenia due to *Providencia rettgeri* bacteremia of unknown origin diagnosed on day 21 after initial influenza diagnosis and was treated with ceftriaxone. Due to persistent thrombocytopenia and asthenia evolving for months attributed to a probable thrombotic microangiopathy, the patient was eventually transferred to our institution. Two days after admission and 33 days after the first influenza-positive test, an NPS was collected because of elevated inflammatory markers and was positive for influenza A with a Ct value of 28. A diagnosis of prolonged viral shedding was made, and oseltamivir monotherapy was resumed for 10 additional days. Treatment was eventually halted at day 43 after initial diagnosis, in the absence of residual symptomatology. On day 52 post-influenza diagnosis, in front of a new onset of fever, a combination therapy of oseltamivir for 10 days and baloxavir every 72 to 96 hours for a total of four doses was resumed. *Providencia rettgeri* was again isolated in the blood cultures and treated with antibiotics. Despite the administration of eculizumab, no improvement was observed regarding the thrombotic microangiopathy, and the patient expired on day 78 post-influenza diagnosis. No effect of influenza treatment on viral load could be observed on further testing. Retrospective sequencing of the first NPS sample collected at our institution, 33 days after the first positive test and after a first oseltamivir treatment course, revealed the presence of the 223R mutation in the NA gene, known to confer reduced inhibition to NAIs, including oseltamivir, zanamivir, and peramivir. On a subsequent NPS sample performed 12 days later, an additional 247G oseltamivir resistance mutation was retrieved.

Patient 4 was treated with an allogeneic HCT for acute myeloid leukemia (AML) 6 months prior to influenza infection. He was quickly diagnosed with AML relapse, which required chemotherapy treatment with 5-azacytidine and venetoclax, leading to persistent neutropenia. One month before the influenza infection, the patient completed a 10-day treatment course of nirmatrelvir/ritonavir for mild COVID-19, leading to SARS-CoV-2 clearance. A few days later, a respiratory syncytial virus (RSV) infection was confirmed on NPS testing. Given a documented LRT involvement on chest CT scan marked by new onset of oxygen requirement, a combination therapy of intravenous immunoglobulins and ribavirin was started 5 days prior to influenza diagnosis. While the symptomatology initially improved, the patient developed new abrupt onset of fever and dyspnea. Testing confirmed an additional influenza A (H3N2) nosocomial infection. Oseltamivir was quickly added to ribavirin, and baloxavir was administered in combination on days 4, 18, and 24 after influenza diagnosis. Viral clearance of influenza was eventually documented on day 36 after symptom onset, while RSV was still detected, yet with a high Ct value (35) on the NPS. Dyspnea resolved, and the patient could be discharged home but died on day 107 after influenza diagnosis due to the underlying hematological disease. Retrospective testing revealed the progressive emergence over time of the I38T (PA) mutation associated with resistance to baloxavir and the R292K (NA) mutation, associated with oseltamivir and zanamivir cross-resistance.

Patient 5 experienced a second relapse of acute lymphoid leukemia 1 month before documented influenza infection, after having received a second allogeneic HCT 2 years previously. She presented with acute respiratory failure progressively evolving over 5 days, in the setting of agranulocytosis post-chemotherapy by gemtuzumab ozogamicin and venetoclax salvage therapy. An NPS was positive for influenza B virus, and combination therapy of oseltamivir and baloxavir was started upon hospitalization. With regard to the poor hematological prognosis, the patient decided to withdraw from intensive care and died at day 9 of hospitalization after initial improvement of respiratory distress. No follow-up NPS to document viral evolution was sampled in this context.

Patient 6 was known for an allogeneic HCT for AML 30 months prior to hospitalization, with complete remission. At the time of influenza infection, the patient was severely immunosuppressed with high-dose steroids, ciclosporin, and imatinib for a severe chronic cutaneous sclerodermic GVHD. He developed progressive dyspnea without other respiratory symptoms or fever, associated with bilateral lung infiltrates suggestive of bronchiolitis obliterans increasing in size over a 3-week period. Bronchoalveolar lavage was positive for influenza A, while the NPS tested negative. Combination therapy of oseltamivir for 5 days with one dose of baloxavir was administered, with limited effect on the symptomatology. The radiological pattern improved after antiviral treatment and diuresis. As the influenza virus was only detected in the lower respiratory tract, no follow-up sample could be collected to document viral clearance.

## DISCUSSION

We describe six highly immunocompromised hematology patients infected with influenza and treated with a combination therapy of an orally administered NAI (oseltamivir) and baloxavir. Four out of six patients died within 3 months of their influenza diagnosis. Prognosis was mainly driven by their underlying disease, their immunosuppression, and additional superinfections. Indeed, this population frequently presents with additional bacterial or fungal superinfections (17), and influenza infection has been shown to be more severe in patients with GVHD after HCT (18). Notably, three out of six patients were diagnosed with an invasive pulmonary fungal infection; five out of six had at least one bacterial infection, while three out of six patients were actively treated for GVHD; one had a relapse of their underlying hematologic malignancy; and another patient was diagnosed with thrombotic microangiopathy requiring additional immunosuppression. Moreover, influenza infection has been associated with organ rejection in transplant patients (19).

Influenza treatment recommendations in severely immunocompromised patients are mainly based on expert opinion (4, 20). Early initiation of antiviral treatment in this population, as in the general population, has been associated with mortality benefit in observational studies, with most data collected with NAI (mainly oseltamivir) treatment (2). Only two retrospective cohort studies evaluated the benefit of baloxavir treatment compared to oseltamivir in hospitalized patients and concluded that outcomes were similar with regard to time to clinical improvement, hospital discharge, and death (11, 21). Both studies included a few immunocompromised patients, with no more than a handful of HCT patients included.

Immunocompromised hosts may display prolonged viral shedding, as it was the case for three of the six patients presented here, who survived their infection long enough to be repeatedly tested (22). More rapid viral clearance and lower emergence of resistance in severely immunocompromised patients have been demonstrated with double-dose oseltamivir with unclear clinical impact (23). Starting in 2019, we decided to change our influenza treatment institutional protocol and chose not to administer double-dose oseltamivir monotherapy because of its documented absence of survival benefit in a retrospective cohort of immunocompromised hosts, where almost 25% of patients included were HCT recipients (24). Instead, we opted for a combination therapy of oseltamivir and baloxavir, based on its synergistic effect, demonstrated *in vitro* as well as in animal models, and as suggested by the Swiss National Influenza Management Guidelines in this population (25–27). While the FLAGSTONE trial, the only randomized controlled trial assessing the efficacy of combination therapy with oseltamivir and baloxavir, failed to show a benefit, a recent post hoc analysis in patients with severe comorbidities such as diabetes, chronic lung diseases, or immunosuppression suggested a potential survival benefit (13, 14). However, only 30 out of 143 patients were immunocompromised, which precludes definitive conclusions. Several other regimens combining amantadine, ribavirin, or favipiravir with oseltamivir, or administering dual NAIs failed to show a clinical benefit of combination therapy in different settings (28–30). In these studies, when included, immunocompromised individuals represented only a small fraction of the participants. Of note, while ribavirin is documented to have an *in vitro* antiviral activity against influenza viruses, it did not prevent the acquisition nor the development of symptoms or antiviral resistance in patient 4 and, in combination with oseltamivir and baloxavir, did not have an impact on the patient’s viral load (31).

Influenza viruses have a high mutation rate, and a single amino acid mutation can lead to drug resistance. In immunocompetent hosts, this phenomenon is more frequently reported with baloxavir than oseltamivir use (32). Protracted shedding in case of immunosuppression further increases the risk of mutation emergence (33). In addition, as there is no cross-resistance for the two antiviral classes, one molecule can be used as a salvage therapy in case of resistance (34). Both oseltamivir and baloxavir influenza-resistant strains can be fit and transmit well (35, 36). Combining different drugs may limit the development of new mutations, and in animal models, baloxavir administration still offered a protective effect on oseltamivir resistance emergence, even in case of pre-existing baloxavir resistance (37). In two of our patients with protracted shedding, the combination therapy did not preclude the development of resistance. Additional data are required to conclude on the benefit of the combination therapy on viral clearance and resistance development in this population, as we only report on a small number of patients, without a control group. An ongoing clinical trial may help to fill in this gap (ClinicalTrials.gov ID: NCT04712539).

Several guidelines on influenza management in immunocompromised individuals suggest administering a prolonged NAI treatment course in case of protracted shedding, some recommending monitoring viral resistance by repeated testing (4, 12, 38, 39). Of note, longer oseltamivir treatment courses are well tolerated, up to 100 days (40). Fewer data exist on repeated baloxavir doses (13). Some of our patients received up to five sequential doses of baloxavir every 72 to 96 hours, and treatment was well tolerated. Of note, while baloxavir is usually safe with few documented adverse events, a larger sample size is required to draw definitive conclusions.

The main limitations of this report are the low number of included patients, the lack of a control group, and the heterogeneity in administered treatment, which preclude drawing definitive conclusions on the effect of additional baloxavir therapy in this population.

In conclusion, combination therapy of oseltamivir and baloxavir, with prolonged treatment courses, was well tolerated in six severely immunocompromised HCT patients. While such treatment led to clinical improvement or viral clearance in most of them, clinical outcomes were mainly determined by their underlying severe conditions. For now, clinical management of influenza in immunocompromised hosts still relies on expert opinion. More data are needed to draw definitive conclusions on the best treatment of HCT patients infected with influenza, and an ongoing clinical trial on combination therapy (ClinicalTrials.gov ID: NCT04712539) may help fill this gap.
